# Association of Urine Metanephrine Levels with CardiometaBolic Risk: An Observational Retrospective Study

**DOI:** 10.3390/jcm10091967

**Published:** 2021-05-04

**Authors:** Mirko Parasiliti-Caprino, Chiara Obert, Chiara Lopez, Martina Bollati, Fabio Bioletto, Chiara Bima, Filippo Egalini, Alessandro Maria Berton, Nunzia Prencipe, Fabio Settanni, Valentina Gasco, Giulio Mengozzi, Ezio Ghigo, Mauro Maccario

**Affiliations:** 1 Endocrinology, Diabetes and Metabolism, Department of Medical Sciences, City of Health and Science University Hospital, University of Turin, 10126 Turin, Italy; chiara.obert@unito.it (C.O.); chiara.lopez@fastewebnet.it (C.L.); bollati.martina@gmail.com (M.B.); fabio.bioletto@unito.it (F.B.); chiara.bimetta@gmail.com (C.B.); filippoegalini@gmail.com (F.E.); alessandro.m.berton@gmail.com (A.M.B.); nunzia.prencipe@gmail.com (N.P.); valentina.gasco@unito.it (V.G.); ezio.ghigo@unito.it (E.G.); mauro.maccario@unito.it (M.M.); 2 Clinical Biochemistry Laboratory, City of Health and Science University Hospital, 10126 Turin, Italy; fabio.settanni@unito.it (F.S.); giulio.mengozzi@unito.it (G.M.)

**Keywords:** catecholamine, adrenaline, noradrenaline, adrenergic regulation, cardiovascular system, cardiovascular risk

## Abstract

No research has explored the role of catecholamine metabolites in the stratification of cardiovascular risk. We aimed to evaluate the relationship between urine metanephrines and cardiometabolic risk/complications. In this retrospective cross-sectional study, we collected the data of 1374 patients submitted to the evaluation of urine metanephrines at the City of Health and Science University Hospital of Turin between 2007 and 2015, mainly for investigating the suspicion of secondary hypertension or the secretion of an adrenal lesion. The univariate analysis showed associations between metanephrines and cardiometabolic variables/parameters, particularly considering noradrenaline metabolite. At univariate regression, normetanephrine was associated with hypertensive cardiomyopathy (OR = 1.18, 95% CI 1.11–1.25; *p* < 0.001) and metabolic syndrome (OR = 1.11, 95% CI 1.03–1.20; *p* = 0.004), while metanephrine was associated with hypertensive cardiomyopathy (OR = 1.23, 95% CI 1.06–1.43; *p* = 0.006) and microalbuminuria (OR = 1.30, 95% CI 1.03–1.60; *p* = 0.018). At multivariate regression, considering all major cardiovascular risk factors as possible confounders, normetanephrine retained a significant association with hypertensive cardiomyopathy (OR = 1.14, 95% CI 1.07–1.22; *p* < 0.001) and metabolic syndrome (OR = 1.10, 95% CI 1.02–1.19; *p* = 0.017). Moreover, metanephrine retained a significant association with the presence of hypertensive cardiomyopathy (OR = 1.18, 95% CI 1.01–1.41; *p* = 0.049) and microalbuminuria (OR = 1.34, 95% CI 1.03–1.69; *p* = 0.019). The study showed a strong relationship between metanephrines and cardiovascular complications/metabolic alterations. Individuals with high levels of these indirect markers of sympathetic activity should be carefully monitored, and they may benefit from an aggressive treatment to reduce the cardiometabolic risk.

## 1. Introduction

Noradrenaline is the principal transmitter of most sympathetic postganglionic fibers and of certain tracts in the central nervous system; dopamine is the predominant transmitter of the extrapyramidal system and of several mesocortical and mesolimbic neuronal pathways and adrenaline is the major hormone of the adrenal medulla. Collectively, these three amines are called catecholamines (CAs) and are derived from the tyrosine metabolism. CAs are stored in vesicles and, particularly in the adrenal medulla, in chromaffin granules. In adults, adrenaline accounts for about 80% of the CAs of the adrenal medulla, with noradrenaline making up most of the remainder [1]. The CAs are adaptive and maladaptive stress hormones. In the classic “fight or flight” mechanism, they activate behavioral and physiological processes that facilitate the overcoming of stress. “Fight or flight” is but one segment of the responses comprising the “general adaptation syndrome”. Feedback processes cooperatively or successively work to sustain homeostasis or “sameness”. For instance, challenged by a physical stressor, an organism responds to the threat either by fighting and prevailing or accepting defeat and fleeing in avoidance. The biological response mounted to combat stress is an adaptive process [2].

In the pathologic context, an excessive CA secretion is typical of the chromaffin tissue tumors, which reflects in blood pressure (BP) elevation, tachycardia, anxiety, pallor, sweating, and headache. Catechol-O-methyltransferase (COMT) catalyzes the O-methylation of the 3-hydroxyl group of most catechols. The O-methylated derivatives of dopamine, noradrenaline, and adrenaline are 3-methoxytyramine, normetanephrine, and metanephrine, respectively. The term “metanephrines” refers to the latter two compounds [3]. Nowadays, it is well recognized that urine and plasma metanephrines are the preferred markers for diagnosis and follow-up of pheochromocytoma and paraganglioma (PPGL) [4,5]. Moreover, these metabolites allow the correct determination of tumor secretive phenotypes, which is useful in the evaluation of PPGL associated risk of recurrence [6]. For the correct interpretation of test results, one has to consider appropriate reference intervals, cut-off values, pretest probability of disease, and the extent of elevation over the upper cut-off value. Nevertheless, in situations of borderline positive test results and low probability of a tumor, a wait-and-retest approach can illuminate increased likelihood of an enlarging small tumor when mild initial elevations are followed by continued increases during follow-up. Endocrine Society guidelines [4] recommend that all positive results should be followed up.

It has been suggested that metanephrines can be considered markers of the whole sympathetic system activity [7], even if they adduce slightly different information than CAs. In fact, metanephrines derive from non-neuronal sources (extra-neuronal and adrenomedullary pathways) because sympathetic nerves contain monoamine oxidase (MAO), but not COMT, and the intraneuronal metabolism of noradrenaline leads to production of the deaminated metabolite 3,4-dihydroxyphenylglycol (DHPG), but not the O-methylated metabolite, normetanephrine [1,8].

Therefore, we hypothesized that the elevation of metanephrines in patients without the evidence of PPGL could be useful in the stratification of cardiovascular (CV) risk and be associated with cardiometabolic complications. To our knowledge, no studies in the literature have explored this issue. To address the potential causes of the disparate findings related to physiologic variation of sympathetic activity, we used an approach characterized by (1) the collection of a large sample size, (2) the evaluation of risk scores as the assessment tool for CV risk, and (3) the application of statistical tools to avoid potential sources of bias. The aim of this study was to determine if there is an association between metanephrine levels and CV risk/cardiometabolic complications.

## 2. Materials and Methods

### 2.1. Design and Study Population

The present study is a retrospective cross-sectional analysis of all consecutive patients referred to the City of Health and Science University Hospital of Turin between September 2007 and September 2015, with the suspicion of PPGL. The study followed the STROBE statement for reporting observational studies [9]. Exclusion criteria were: age < 18 years old, previous cardiovascular disease (CVD), chronic heart failure, arrhythmias, causes of secondary hypertension (obstructive sleep apnoea, primary aldosteronism, renal artery stenosis, pheochromocytoma/paraganglioma, primary hyperparathyroidism, autonomous cortisol secretion, or overt hypercortisolism), renal insufficiency with estimated glomerular filtration rate (eGFR) < 30 mL/min, psychiatric illness, liver cirrhosis, chronic diseases with major organ involvement, excessive alcohol ingestion, current assumption of sympathomimetics, and cocaine and other drugs commonly affecting the measurement of metanephrines (Acetaminophen, Labetalol, Sotalol, tricyclic antidepressants, Buspirone, Phenoxybenzamine, MAO inhibitors, Sulphasalazine and Levodopa). For patients with hormonal values over the upper cut-off, accurate endocrinological evaluation (supplemental metanephrine determinations, adding also the quantification of chromogranin A, neuron specific enolase and also performing CT/MRI with contrast of the abdomen or specific functional imaging test such as ^123^I-MIBG Scintigraphy, PET with ^18^F-FDOPA, or ^68^Ga-DOTATOC, where appropriate) and follow-up were performed. Patients affected by PPGL and carriers of genetic mutations were excluded, checking data from the registry of the Piedmont Oncological Network and considering also at least 5 years of follow-up to avoid the risk of including small PPGL with mild/non-secretive phenotype.

Data were collected within prospective registries and analyzed retrospectively. The study was performed in accordance with the guidelines in the Declaration of Helsinki and approved by the Ethics Committee of City of Health and Science University Hospital of Turin (No. 0035241). Written informed consent was obtained from all enrolled patients (ClinicalTrials.gov No. 04495231).

The methods and materials used in this analysis are available to any researcher for the purposes of reproducing the results or replicating the procedures.

### 2.2. Clinical and Biochemical Investigations

Personal data; familial history of arterial hypertension, diabetes mellitus (DM), and early cardiovascular event; reason for CA metabolites dosage (hypertensive crisis, suspicion of secondary hypertension, adrenal lesion, or resistant hypertension); BP values; serum levels of glucose; lipid profile; and sodium, potassium, creatinine, and 24 h urinary metanephrine levels were collected. Measurement of CA metabolites was performed on single or double 24 h urine collection. Patients were advised to empty bladder into the toilet when they got up in the morning, without including this urine in the collection but collecting the remainder of the 24 h period. The test ended 24 h after the first (uncollected) specimen, keeping the urine container refrigerated or in a cool place during the collection, until return to the laboratory. In the case of two or more dosages, we considered the value of the first collection.

Cardiovascular risk was estimated using a risk calculator (Framingham risk score [10], Progetto CUORE [11], and SCORE [12]). The office BP values were collected according to guidelines [13].

### 2.3. Evaluation of Metabolic Syndrome and Organ Damage (OD)

Metabolic syndrome (MS) was defined according to the ATP III criteria [14]. OD was defined according to the 2018 ESC/ESH guidelines. The eGFR was estimated using the CKD-EPI formula, and microalbuminuria was defined between 30–300 mg/24 h or by an albumin to creatinine ratio of 30–300 mg/g. Left ventricular mass index was calculated with the formula: 0.8 x 1.04 x [(interventricular septum + left ventricular internal diameter + inferolateral wall thickness)^3^ - left ventricular internal diameter^3^] + 0.6 gr; left ventricular hypertrophy, assessed by echocardiography, was defined by a left ventricular mass index >115 g/m^2^ for men and >95 g/m^2^ for women; and for obese subjects left ventricular mass was indexed for height (left ventricular hypertrophy was defined by a left ventricular mass index >50 g/m^2.7^ for men and >47 g/m^2.7^ for women) [13].

### 2.4. Analytical Methods

As previously described [15], 24-h urinary metanephrines (µg/day) were measured by chromatographic determination on an isocratic high-performance liquid chromatography (HPLC) system with an electrochemical detector fixed with a potential of 740 mV (Chromsystems Instruments & Chemicals GmbH, Gräfelfing, Germany). Briefly, 1 mL of 24 h urine samples were mixed with 100 µL Internal Standard and poured in sealable hydrolysis tubes that were incubated for 30 min at 90–100 °C in a water bath. Then a neutralization buffer was added to the samples, and all of the neutralized urines were applied to sample clean up columns for a solid-phase extraction that were successively mixed, centrifuged, and washed. Certain amounts (20–50 μL) of eluates were injected into the HPLC system, and the retention times of normetanephrine and metanephrine and Internal Standard were, respectively, 5.5, 7.0, and 8.4 min. Furthermore, limits of quantification, intra-assay, and inter-assay coefficients of variation were, respectively, 5 mg/L, 1.4%, and 2.7% for normetanephrine and 11 mg/L, 1.8%, and 2.8% for metanephrine.

### 2.5. Statistical Analysis

Baseline characteristics of all patients included in the analysis are summarized using mean and standard deviation (SD) after the analysis of each variable/parameter through the quantile-quantile (Q-Q) plot, considering the large sample size. Binary and categorical data were reported using percent values. In the descriptive statistics, the sample was divided according to the tertiles of normetanephrine and metanephrine. Between groups, differences in personal and clinical features were evaluated by one-way ANOVA for continuous variables and chi-square test or Fisher’s exact test for categorical variables, as appropriate.

Associations between the levels of normetanephrine/metanephrine and the presence of cardiometabolic/renal complications (hypertensive cardiomyopathy, metabolic syndrome, eGFR < 60 mL/min/1.73 m^2^, and albuminuria) were assessed through univariate and multivariate logistic regressions, considering as possible confounders all the risk factors known to be possibly related to adverse cardiovascular or renal outcomes. In this analysis, metanephrines were considered as continuous variables, and the coefficients of the models (with correspondent odds ratios) were computed by considering 100 μg/die as their unitary increase. A *p*-value < 0.05 was considered statistically significant. Statistical analysis was performed using R 3.5.3 (R Core Team, R Foundation for Statistical Computing, Vienna, Austria, 2019).

## 3. Results

**Figure 1:**
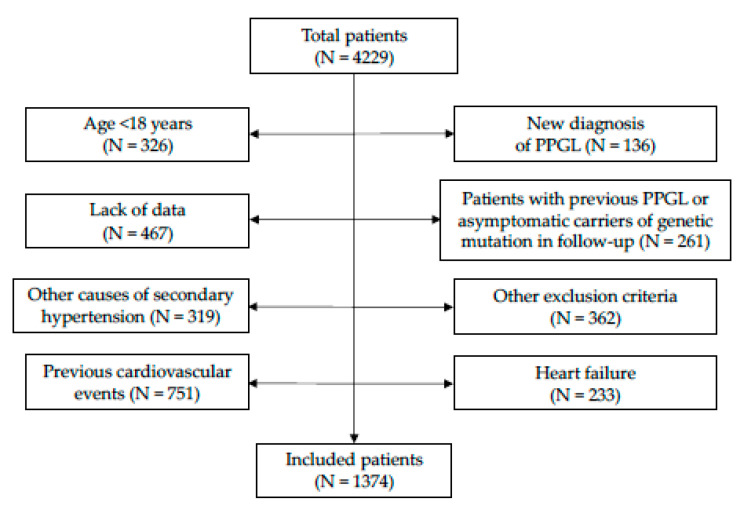
Study flow-chart. Abbreviations: PPGL, pheochromocytoma or paraganglioma.

The study, as shown in Figure 1, enrolled 1374 patients: 571 males (41.6%) and 803 females (58.4%). The series was divided into tertiles of normetanephrine (I tertile: 20.0–232.1 μg/die; II tertile: 232.7–367.0 μg/die; III tertile: 368.0–2300 μg/die) and metanephrine (I tertile: 10.0–73.0 μg/die; II tertile: 73.2–123.3 μg/die; III tertile: 123.8–851.5 μg/die). In the univariate analysis, patients with higher levels of urine normetanephrine proved to be older (*p* < 0.001), had higher rate of male gender (*p* < 0.001), smoking habit (*p* < 0.001), obesity (*p* = 0.002), arterial hypertension (*p* < 0.001), history of hypertensive crisis (*p* < 0.001), prediabetes/diabetes mellitus (*p* < 0.001), metabolic syndrome (*p* = 0.026), hypertensive cardiomyopathy (*p* < 0.001), higher values of BMI (*p* < 0.001), weight (*p* < 0.001), office systolic BP (SBP, *p* = 0.002), office diastolic BP (DBP, *p* = 0.014), cardiovascular risk scores (Framingham risk score *p* < 0.001; SCORE, *p* = 0.009; Progetto CUORE, *p* < 0.001), triglycerides (*p* = 0.001), and lower levels of left ventricular ejection fraction (*p* = 0.003), compared to patients with normetanephrine in the I-II tertiles.

Regarding the drug therapy, patients with high levels of normetanephrine had a higher rate of treatment with lipid lowering drugs (*p* = 0.004), β-blockers (*p* = 0.043), α-blockers (*p* = 0.008), angiotensin II receptor blockers (ARB, *p* = 0.026), thiazides (-like) diuretics (*p* = 0.011), calcium channel blockers (CCB, *p* = 0.037), and loop diuretics (*p* = 0.016), compared to patients with normetanephrine in the I–II tertiles. While individuals with higher values of urine metanephrine had a higher rate of male gender (*p* < 0.001), smoking habit (*p* < 0.001), hypertensive crisis (*p* < 0.001), higher levels of Framingham risk score (*p* = 0.001), serum creatinine (*p* = 0.019), lower values of BMI (*p* < 0.001), weight (*p* = 0.005), and therefore lower rate of obesity (*p* = 0.004), compared to patients with metanephrines in the I–II tertiles. No differences were found in the remaining variables/parameters (Table 1).

### Univariate and Multivariate Logistic Regressions

In the current study, we analyzed urine metanephrines as a possible independent variable associated with cardiometabolic and renal complications, considering all other common risk factors as potential confounders of these associations.

At univariate analysis, normetanephrine proved to be associated to hypertensive cardiomyopathy (OR = 1.18, 95% CI 1.11–1.25; *p* < 0.001) and metabolic syndrome (OR = 1.11, 95% CI 1.03–1.20; *p* = 0.004); conversely, no significant association with microalbuminuria and with eGFR < 60 mL/min/1.73 m^2^ was found (data not shown).

At multivariate analysis, normetanephrine retained a statistically significant association with:

- Hypertensive cardiomyopathy (OR = 1.14, 95% CI 1.07–1.22; *p* < 0.001); other covariates that showed to be independently associated with the outcome in this model were male gender (OR = 1.44, 95% CI 1.06–1.97; *p* = 0.020), age (OR = 1.01, 95% CI 1.01–1.03; *p* = 0.003), smoking habit (OR = 1.62, 95% CI 1.19–2.19; *p* = 0.002), BMI (OR = 1.03, 95% CI 1.00–1.06; *p* = 0.050), and number of antihypertensive drugs (OR = 1.60, 95% CI 1.37–1.87; *p* < 0.001) (Table 2);
- Metabolic syndrome (OR = 1.10, 95% CI 1.02–1.19; *p* = 0.017), after correction for gender, age, smoking habit, familial history of CVD, number of antihypertensive drugs (OR = 1.28, 95% CI 1.12–1.66; *p* < 0.001), and eGFR (Table 3).

Urine normetanephrine was not significantly associated with the presence of microalbuminuria (Table 4) and impaired renal function (Table 5).

Regarding adrenaline metabolites, at univariate logistic regression, metanephrine proved to be associated with hypertensive cardiomyopathy (OR = 1.23, 95% CI 1.06–1.43; *p* = 0.006) and microalbuminuria (OR = 1.86, 95% CI 1.03–1.60; *p* = 0.018); conversely, no significant associations with metabolic syndrome or eGFR < 60 mL/min/1.73 were found (data not shown).

At multivariate analysis, metanephrine retained a statistically significant association with:

- Hypertensive cardiomyopathy (OR = 1.18, 95% CI 1.01–1.41; *p* = 0.049); other covariates that showed to be independently associated with the outcome were age (OR = 1.02, 95% CI 1.01–1.03; *p* = 0.002), smoking habit (OR = 1.62, 95% CI 1.20–2.20; *p* = 0.002), BMI (OR = 1.03, 95% CI 1.01–1.06; *p* = 0.018), and number of antihypertensive drugs (OR = 1.60, 95% CI 1.37–1.87; *p* < 0.001) (Table 2);
- Microalbuminuria (OR = 1.34, 95% CI 1.03–1.69; *p* = 0.019), considering gender, age, smoking habit, familial history of CVD, BMI, office SBP, office DBP (OR = 1.03, 95% CI 1.00–2.06; *p* = 0.036), DM, eGFR (OR = 0.97, 95% CI 0.96–0.98; *p* < 0.001), and treatment with ACEi/ARB as covariates (Table 4).

Urine metanephrine was not associated with metabolic syndrome and eGFR < 60 mL/min/1.73 m^2^.

To exclude interferences and potential sources of bias, the series was analyzed with the same statistics after excluding all patients treated with drugs affecting the sympathetic system. The results, shown in the Supplementary Materials (Table S1–S4), were not different from those previously observed for normetanephrine, while the significant results for metanephrine were not confirmed, likely because of the reduction of the statistical power.

## 4. Discussion

The present study demonstrated the association between 24-h urinary metanephrine levels and cardiovascular risk/cardiometabolic complications in a large cohort of patients in primary prevention, screened for the suspicion of secondary hypertension and/or for the assessment of adrenal incidentaloma secretion. Particularly, high levels of normetanephrine were proven to be associated with hypertensive cardiomyopathy and metabolic syndrome, while high metanephrine levels showed an association with hypertensive cardiomyopathy and microalbuminuria. Our study provided evidence on the further role of a simple diagnostic tool for cardiovascular risk stratification, demonstrating the capability of urine metanephrines to indirectly describe sympathetic activity, not only in the diagnosis of PPGL.

Data in the literature suggest that neuroadrenergic influences play a role in the diet-induced thermogenesis [16], predisposing to obesity/overweight, glucose metabolism impairment, metabolic syndrome [17], and in the genesis or progression of arterial hypertension [18], organ damage, and therefore cardiovascular risk [19]. Moreover, a heightened sympathetic activity has been reported in patients with masked uncontrolled hypertension compared with true controlled hypertensives [20] and in patients with refractory hypertension compared to individuals with resistant hypertension [15,21].

Indirect markers of sympathetic activity, mainly plasma and urine CA, have been adopted in some studies, showing conflicting results about the association with cardiometabolic complications, possibly due to methodological issues (small sample size, heterogeneity of the enrolled patients, and retrospective design). Particularly, plasma noradrenaline levels proved to be associated with insulin resistance [22,23], arterial hypertension [23,24,25], and weight gain [24,25]. In the Chinese population, a high urine noradrenaline and low adrenaline excretion have been demonstrated in patients with metabolic syndrome [26], suggesting that there can be dissociations between the sympathetic nervous system and adrenal medullary function, as shown with other reflex responses.

Urine CA were not found to be associated with high levels of blood pressure [27]. Regarding catecholamine metabolites, plasma metanephrines demonstrated a relationship with impaired glucose metabolism/risk of prediabetes [28], left ventricular hypertrophy, and diastolic dysfunction [29]. Coulson et al. [30] observed a correlation between 24-h urinary normetanephrine and measures of SBP variability and hypothesized that sympathetic activity may influence SBP variability. In a large part, these results are consistent with our data, even if the considered studies have a different design and smaller sample size compared to the present study.

In the present research, patients taking drugs commonly affecting the measurement of metanephrines were excluded. Individuals with high normetanephrine levels took more antihypertensive drugs in comparison with patients in the first and second tertile. These differences have been demonstrated for almost all antihypertensive classes, even if not always statistically significant. We would like to emphasize that these results apply to all the commonly prescribed antihypertensive drugs (ACEi/ARB, CCB e thiazide diuretics), and for this reason we considered not significant the potential impact of antihypertensive drugs acting on the sympathetic system (mainly α- and β-blockers) on the levels of metanephrines. Nevertheless, we also repeated the multivariate analyses, excluding patients taking these classes of drugs, and the results were not different from those previously observed for normetanephrine, while the significant results for metanephrine were not confirmed, likely because of the reduction of the statistical power.

In the last few years, the introduction of new techniques (such as whole body and regional noradrenaline spillover to plasma, microneurography, and pharmacological methods) demonstrated the role of sympathetic activity in overweight, obesity [31], metabolic syndrome [32,33], impaired glucose metabolism [34], type 2 diabetes [35], and left ventricular hypertrophy [35,36].

Nevertheless, these tools are not widely available and not simple to apply [37]. Very simple and important clinical data is heart rate (HR), which is a marker of adrenergic overdrive in metabolic syndrome [38] with a limited reliability because this variable is unable to reflect the main metabolic and anthropometric abnormalities characterizing the syndrome. Nevertheless, in a recent article, Grassi et al. [39] provided strong evidence on the association between HR > 80 bpm and sympathetic activation and the correlation with increased cardiovascular risk, as suggested by 2018 ESC/ESH guidelines on arterial hypertension [13].

We think the results of the present study are very important because, for the first time, we have demonstrated the role of 24-h urine metanephrines, traditionally performed for the case detection of PPGL, also in the stratification of cardiovascular risk.

The strengths of this study are the inclusion of a very large cohort, the collection of data from a prospective single center registry, and the high quality of the third-level laboratory that performed the analysis of metanephrines.

This study has some limitations. First of all, the retrospective cross-sectional design, which prevented us from evaluating a causal relationship between metanephrines and CV complications. Second, metanephrines are indirect markers of sympathetic tone; as previously reported, more direct method for the evaluation of sympathetic activity have been developed, but these techniques are not widely available and their application is limited to the in-clinic setting. Third, there is the possibility of having included small and non-secretive PPGL or chromaffin tumors with normal metabolites, which may bias the cardiovascular stratification. However, only a very small number of patients with PPGL do not experience sympathetic related symptoms and/or elevated metanephrines; moreover, PPGL are rare diseases, therefore the potential confounding effect of these misdiagnoses cannot be relevant. Fourth, the methods used for urine metanephrines evaluation measured free plus sulfate-conjugated metabolites, and urine collections have not been corrected for urine creatinine excretion. However, the consistency of the data obtained, even in multivariable models, suggests that these limitations were not critical factors for the main results of the present study. It should be kept in mind that the population of our study is composed of patients with suspicion of PPGL and that, for generalizing these data to the overall population, an ad hoc study might be necessary.

Therefore, if confirmed, our results would introduce a new simple marker of sympathetic activity and cardiovascular risk, able to guide the clinicians into a better stratification of cardiometabolic patients, with the intent to identify those who should be treated more aggressively. A reduction in the sympathetic tone through targeted therapy for the control of BP and HR, or indirectly through the control of the metabolic profile, has demonstrable positive effects beyond BP-related effects. A certain quota of sympathoinhibition may be obtained with weight loss through lifestyle changes or medical/surgical therapy [40,41], sodium restriction, antihypertensive agents [42], baroreflex activation [43], statins, and some classes of antidiabetic drugs [37,44,45,46]. It has still to be demonstrated that patients with high levels of urine metanephrines, and therefore increased cardiovascular risk, also have a high incidence of cardiovascular events. If this hypothesis is confirmed, the correct identification and treatment of patients with heightened sympathetic tone could result in a reduction of cardiovascular events/mortality, possibly by the adoption of specific sympathoinhibitory strategies that, in the last few years, have not been considered as the first-line approach for the treatment of arterial hypertension.

## Figures and Tables

**Table 1:** Distributions of categorical and continuous variables/parameters according to tertiles of normetanephrine and metanephrine levels.

Variables/ Parameters	Overall Data (*n* = 1374)	Normetanephrine				Metanephrine			
		I Tertile	II Tertile	III Tertile	*p*-Value	I Tertile	II Tertile	III Tertile	*p*-Value
Age (years)	54 ± 15	50 ± 15	55 ± 15	56 ± 13	<0.001 *,†	54 ± 16	54 ± 15	53 ± 14	0.729
Male Gender	41.6%	29.7%	40.0%	55.0%	<0.001 *,†,‡	28.5%	39.7%	56.6%	<0.001 *,†,‡
Smoking habit	38.9%	31.7%	40.0%	45.2%	<0.001 *,‡	32.8%	39.3%	44.8%	<0.001 *,†
FH of CVD	12.2%	13.3%	13.3%	10.1%	0.233	13.2%	11.5%	12.0%	0.725
FH of AH	44.0%	46.7%	42.8%	42.3%	0.340	43.0%	43.7%	45.2%	0.788
FH of DM	23.4%	23.4%	24.8%	21.9%	0.594	24.8%	24.3%	21.0%	0.324
Arterial hypertension	86.6%	81.9%	85.9%	92.1%	<0.001 †,‡	87.7%	86.1%	86.0%	0.703
Hypertensive Crisis	9.0%	3.7%	6.5%	16.7%	<0.001 †,‡	5.4%	9.1.%	12.5%	<0.001 *,†
Adrenal Lesion	32.0%	31.2%	31.7%	33.1%	0.818	34.1%	30.0%	31.9%	0.411
Weight (Kg)	74.5 ± 16.4	69.7 ± 15.5	74.9 ± 15.8	78.9 ± 16.5	<0.001 *,†,‡	74.7 ± 17.2	72.6 ± 16.0	76.3 ± 15.7	0.005 ‡
BMI (kg/m^2^)	26.7 ± 5.5	25.3 ± 4.9	27.1 ± 5.8	27.5 ± 5.4	<0.001 *,†	27.5 ± 6.1	26.1 ± 5.4	26.5 ± 4.7	<0.001 *,†
Obesity	20.3%	14.7%	21.8%	24.4%	0.002 *,‡	25.6%	17.3%	18.1%	0.004 *,†
Office SBP (mmHg)	135 ± 17	133 ± 17	135 ± 16	137 ± 19	0.002 †,‡	134 ± 16	136 ± 17	136 ± 19	0.089 *
Office DBP (mmHg)	82 ± 11	81 ± 10	82 ± 11	83 ± 12	0.014 †,‡	82 ± 10	83 ± 11	83 ± 12	0.341
DM	11.3%	9.9%	10.9%	13.0%	0.326	12.2%	11.8%	9.8%	0.487
Prediabetes/ DM	21.2%	16.8%	19.8%	27.0%	<0.001 †,‡	22.0%	22.5%	19.0%	0.369
Associated tumors	29.7%	29.0%	28.1%	32.0%	0.401	30.5%	31.3%	27.3%	0.367
Framingham risk score (%)	7.6 ± 8.3	5.3 ± 6.4	7.9 ± 9.1	9.5 ± 8.7	<0.001 *,†,‡	6.3 ± 6.7	7.8 ± 9.6	8.4 ± 8.3	0.001 *,†
SCORE (%)	3.2 ± 6.7	2.3 ± 4.1	3.3 ± 4.9	3.8 ± 9.4	0.009 *,†	2.9 ± 4.8	3.4 ± 8.2	3.2 ± 6.6	0.701
Progetto Cuore (%)	9.0 ± 11.2	7.1 ± 11.2	9.7 ± 12.5	10.5 ± 11.7	<0.001 *,†	8.6 ± 11.9	9.2 ± 12.5	9.5 ± 11.2	0.592
EF (%)	60 ± 8	61 ± 7	61 ± 6	58 ± 9	0.003 †,‡	62 ± 7	60 ± 7	59 ± 8	0.109
Glucose (mg/dL)	95 ± 26	93 ± 23	96 ± 29	98 ± 24	0.018	95 ± 25	97 ± 28	95 ± 24	0.416
Total Cholesterol (mg/dL)	190.7 ± 45.3	187.3 ± 41.3	192.1 ± 45.6	192.6 ± 48.4	0.220	194.0 ± 45.4	190.3 ± 43.7	187.9 ± 46.6	0.187
Triglycerides (mg/dL)	119.8 ± 58.9	111.4 ± 53.0	120.4 ± 51.1	127.4 ± 69.1	0.001 *,†	118.7 ± 57.0	120.0 ± 67.5	120.9 ± 51.7	0.876
HDLc (mg/dL)	50.3 ± 17.6	51.0 ± 14.6	50.1 ± 18.9	49.9 ± 19.1	0.662	51.1 ± 14.6	49.1 ± 15.0	50.8 ± 22.1	0.291
LDLc (mg/dL)	119.7 ± 39.9	114.6 ± 34.8	119.4 ± 40.1	119.9 ± 40.7	0.060 *,†	121.6 ± 38.2	119.3 ± 40.0	118.0 ± 41.3	0.474
ECG HR (bpm)	75 ± 16	74 ± 15	75 ± 16	75 ± 17	0.423	74 ± 16	75 ± 17	75 ± 16	0.665
Indication for quantification									
*Hypertensive crisis*	8.2%	3.0%	5.8%	15.5%	<0.001 *	5.2%	7.3%	12.1%	0.003
*Suspicion of* *secondary AH*	55.1%	60.6%	56.8%	48.0%		55.4%	58.7%	51.2%	
*Adrenal* *lesion*	24.5%	21.5%	26.2%	25.8%		28.3%	21.9%	23.2%	
*Resistant* *Hypertension*	10.2%	13.1%	8.5%	9.1%		8.7%	11.1%	1.9%	
*Unknown*	2.0%	1.8%	2.7%	1.7%		2.4%	1.0%	2.7%	
Metabolic Syndrome	16.7%	12.3%	18.0%	19.9%	0.026 *,‡	19.4%	15.1%	15.8%	0.282
Microalbuminuria	6.3%	6.8%	5.4%	6.8%	0.625	5.0%	6.4%	7.6%	0.249
Creatinine (mg/dL)	0.90 ± 0.57	0.90 ± 0.75	0.85 ± 0.33	0.94 ± 0.54	0.072 ‡	0.89 ± 0.68	0.84 ± 0.33	0.95 ± 0.62	0.019 ‡
eGFR (CKD-EPI, mL/min/1.73m^2^)	93 ± 31	94 ± 35	93 ± 27	92 ± 29	0.507 †,‡	92 ± 30	92 ± 30	94 ± 32	0.401
Hypertensive cardiomyopathy	18.0%	9.0%	17.4%	27.7%	<0.001 *,†,‡	16.4%	17.3%	20.3%	0.271
Lipid-lowering drugs	13.5%	9.6%	13.7%	17.1%	0.004 *,‡	14.5%	12.6%	13.3%	0.707
No. of antihypertensive drugs	1.30 ± 1.25	1.09 ± 1.19	1.29 ± 1.22	1.53 ± 1.30	<0.001 *,†,‡	1.37 ± 1.30	1.28 ± 1.26	1.24 ± 1.19	0.776
β-blockers	20.3%	17.3%	19.8%	23.9%	0.043 ‡	22.7%	17.5%	20.7%	0.143
α-blockers	8.6%	6.3%	7.6%	11.8%	0.008 †,‡	8.9%	9.3%	7.6%	0.654
α-2 agonists	1.9%	2.2%	2.2%	1.3%	0.541	1.7%	2.2%	1.7%	0.832
Methyldopa	0.6%	1.1%	0.2%	0.4%	0.193	0.9%	0.7%	0.2%	0.420
ACEi	20.6%	17.7%	20.7%	23.5%	0.100 ‡	19.2%	19.5%	23.1%	0.259
ARB	23.0%	19.0%	23.5%	26.5%	0.026 ‡	22.7%	23.0%	23.4%	0.970
Thiazide (-like) diuretics	19.9%	15.8%	20.4%	23.7%	0.011 ‡	17.3%	21.2%	21.4%	0.208
MRA	3.7%	3.1%	2.8%	5.3%	0.100	3.5%	2.7%	5.0%	0.158
CCB	29.4%	25.6%	29.3%	33.3%	0.037 ‡	32.4%	29.0%	26.9%	0.177
Amiloride	2.1%	0.9%	2.2%	3.1%	0.061 ‡	2.2%	1.8%	2.2%	0.883
Loop diuretics	7.7%	5.7%	6.7%	10.5%	0.016 †,‡	8.0%	6.0%	9.0%	0.225

Abbreviations: ACEi, angiotensin converting enzyme inhibitors; AH, arterial hypertension; ARB, angiotensin II receptor blockers; BMI, body mass index; CCB, calcium channel blockers; CKD-EPI, chronic kidney disease epidemiology collaboration; CVD, cardiovascular disease; DBP, diastolic blood pressure; DM, diabetes mellitus; EF, ejection fraction; eGFR, estimated glomerular filtration rate; FH, familial history; HDL, high density lipoprotein; HR, heart rate; LDLc, low density lipoprotein calculated; MRA, mineralocorticoid receptor antagonist; SBP, systolic blood pressure. * Significant difference between I tertile and II tertile. † Significant difference between I tertile and III tertile. ‡ Significant difference between II tertile and III tertile.

**Table 2:** Logistic regression analysis on the association of metanephrines and covariates with the presence of hypertensive cardiomyopathy (ORs of normetanephrine and metanephrine are calculated for a unit of increase of 100 μg/die).

Covariates	Hypertensive Cardiomyopathy					
	OR	95% CI	*p*-Value	OR	95% CI	*p*-Value
Gender	1.44	1.06–1.97	0.020	1.52	1.11–2.07	0.769
Age	1.01	1.01–1.03	0.003	1.02	1.01–1.03	0.002
Smoking habit	1.62	1.19–2.19	0.002	1.62	1.20–2.20	0.002
FH of CVD	0.95	0.60–1.48	0.833	0.90	0.57–1.39	0.637
BMI	1.03	1.00–1.06	0.050	1.03	1.01–1.06	0.018
SBP	1.00	0.99–1.01	0.582	1.00	0.99–1.02	0.467
DBP	1.01	0.99–1.03	0.222	1.01	0.99–1.03	0.212
DM	1.30	0.83–1.99	0.243	1.36	0.88–2.09	0.156
No. of antihypertensive drugs	1.60	1.37–1.87	<0.001	1.60	1.37–1.87	<0.001
ACEi/ARB	0.94	0.64–1.38	0.742	0.93	0.63–1.37	0.714
*Normetanephrine*	1.14	1.07–1.22	<0.001	-	-	-
*Metanephrine*	-	-	-	1.18	1.01–1.41	0.049

Abbreviations: ACEi/ARB, angiotensin converting enzyme inhibitors or angiotensin II receptor blockers; BMI, body mass index; CI, confidence interval; CVD, cardiovascular disease; DBP, diastolic blood pressure; DM, diabetes mellitus; FH, family history; OR, odds ratio; SBP, systolic blood pressure.

**Table 3:** Logistic regression analysis on the association of metanephrines and covariates with the presence of metabolic syndrome (ORs of normetanephrine and metanephrine are calculated for a unit of increase of 100 μg/die).

Covariates	Metabolic Syndrome					
	OR	95% CI	*p*-Value	OR	95% CI	*p*-Value
Gender	1.07	0.71–1.62	0.800	1.07	0.75–1.53	0.702
Age	1.00	0.98–1.01	0.608	1.00	0.99–1.01	0.697
Smoking habit	1.21	0.61–1.38	0.281	1.25	0.88–1.77	0.209
FH of CVD	1.40	0.87–2.20	0.157	1.32	0.82–2.08	0.234
No. of antihypertensive drugs	1.28	1.12–1.66	<0.001	1.30	1.14–1.49	<0.001
eGFR	1.00	0.45–1.27	0.920	1.00	0.99–1.01	0.999
*Normetanephrine*	1.10	1.02–1.19	0.017	-	-	-
*Metanephrine*	-	-	-	0.89	0.71–1.10	0.320

Abbreviations: CI, confidence interval; CVD, cardiovascular disease; eGFR, estimated glomerular filtration rate; FH, family history; OR, odds ratio.

**Table 4:** Logistic regression analysis on the association of covariates with the presence of microalbuminuria (ORs of metanephrine are calculated for a unit of increase of 100 μg/die).

Covariates	Microalbuminuria					
	OR	95% CI	*p*-Value	OR	95% CI	*p*-Value
Gender	1.75	1.03–2.97	0.038	1.10	0.96–2.77	0.689
Age	0.98	0.97–1.00	0.101	1.63	0.97–1.00	0.109
Smoking habit	1.27	0.76–2.13	0.360	1.24	0.74–2.08	0.411
FH of CVD	1.50	0.71–2.91	0.256	1.55	0.74–2.03	0.216
BMI	0.96	0.91–1.01	0.158	0.97	0.91–1.02	0.203
SBP	0.99	0.97–1.01	0.293	0.99	0.97–1.01	0.268
DBP	1.03	1.00–1.06	0.037	1.03	1.00–2.06	0.036
DM	1.54	0.71–3.09	0.245	1.62	0.74–3.25	0.198
eGFR	0.97	0.96–0.98	<0.001	0.97	0.96–0.98	<0.001
ACEi/ARB	1.07	0.64–1.80	0.793	1.06	0.63–1.79	0.811
*Normetanephrine*	1.05	0.94–1.15	0.382	-	-	-
*Metanephrine*	-	-	-	1.34	1.03–1.69	0.019

Abbreviations: ACEi/ARB, angiotensin converting enzyme inhibitors or angiotensin II receptor blockers; BMI, body mass index; CI, confidence interval; CVD, cardiovascular disease; DBP, diastolic blood pressure; DM, diabetes mellitus; eGFR, estimated glomerular filtration rate; FH, family history; OR, odds ratio; SBP, systolic blood pressure.

**Table 5:** Logistic regression analysis on the association of metanephrines and covariates with the presence of eGFR < 60 mL/min/1.73 m^2^ (ORs of normetanephrine and metanephrine are calculated for a unit of increase of 100 μg/die).

Covariates	eGFR < 60 mL/min/1.73 m^2^					
	OR	95% CI	*p*-Value	OR	95% CI	*p*-Value
Gender	1.13	0.71–1.63	0.725	1.14	0.75–1.72	0.542
Age	1.04	1.02–1.06	<0.001	1.04	1.02–1.06	<0.001
Smoking habit	0.91	0.62–1.39	0.669	0.93	0.61–1.39	0.716
FH of CVD	1.01	0.54–1.79	0.977	0.99	0.52–1.75	0.967
BMI	0.99	0.95–1.03	0.486	0.99	0.92–1.02	0.513
SBP	1.01	1.00–1.03	0.060	1.01	1.00–1.03	0.050
DBP	0.98	0.96–1.00	0.127	0.98	0.96–1.00	0.121
DM	1.28	0.73–2.18	0.369	1.28	0.73–2.18	0.370
No. of antihypertensive drugs	1.28	1.09–1.49	0.002	1.29	1.10–1.50	0.001
*Normetanephrine*	1.05	0.95–1.14	0.320	-	-	-
*Metanephrine*	-	-	-	0.95	0.72–1.20	0.663

Abbreviations: BMI, body mass index; CI, confidence interval; CVD, cardiovascular disease; DBP, diastolic blood pressure; DM, diabetes mellitus; eGFR, estimated glomerular filtration rate; FH, family history; OR, odds ratio; SBP, systolic blood pressure.

## Data Availability

The methods and materials used in this analysis are available to any researcher for the purposes of reproducing the results or replicating the procedures.

## Supplementary Materials

The following are available online at https://www.mdpi.com/article/10.3390/jcm10091967/s1: Table S1 (logistic regression analysis on the association of metanephrines and covariates with presence of hypertensive cardiomyopathy, after excluding patients taking drugs acting on the sympathetic system); Table S2 (logistic regression analysis on the association of metanephrines and covariates with presence of metabolic syndrome, after excluding patients taking drugs acting on the sympathetic system); Table S3 (logistic regression analysis on the association of covariates with presence of microalbuminuria, after excluding patients taking drugs acting on the sympathetic system; Table S4 (logistic regression analysis on the association of metanephrines and covariates with presence of eGFR<60 mL/min/1.73 m^2^, after excluding patients taking drugs acting on the sympathetic system).
