# Supplementary material for: Association of Urine Metanephrine Levels with CardiometaBolic Risk: An Observational Retrospective Study
Source: J Clin Med. 2021 May 4;10(9):1967. doi: 10.3390/jcm10091967 (PMC8125207; doi:10.3390/jcm10091967)
Supplement: Supplementary file 1 [file jcm-10-01967-s001.zip › jcm-1182372-supplementary.pdf]

Covariates	Hypertensive cardiomyopathy					
	**OR**	**95% CI**	**p-value**	**OR**	**95% CI**	**p-value**
Gender	1.17	0.79-1.74	0.430	1.23	0.83-1.83	0.296
Age	1.02	1.00-1.03	0.029	1.02	1.00-1.03	0.022
Smoking habit	2.00	1.36-2.95	<0.001	2.02	1.37-2.98	<0.001
FH of CVD	1.17	0.65-2.02	0.587	1.12	0.62-1.93	0.701
BMI	1.04	1.01-1.08	0.016	1.05	1.01-1.08	0.010
SBP	1.01	0.99-1.02	0.222	1.01	1.00-1.02	0.154
DBP	1.01	0.99-1.04	0.192	1.01	0.99-1.04	0.202
DM	1.65	0.94-2.82	0.074	1.66	0.95-2.84	0.068
No. of antihypertensive drugs	1.40	1.06-1.84	0.015	1.41	1.07-1.85	0.013
ACEi/ARB	1.22	0.73-2.08	0.449	1.21	0.72-2.04	0.483
Normetanephrine	1.09	1.00-1.18	0.035	-	-	-
Metanephrine	-	-	-	1.07	0.85-1.33	0.581

Table S1. Logistic regression analysis on the association of metanephrines and covariates with presence of hypertensive cardiomyopathy, after excluding patients taking drugs acting on the sympathetic system (ORs of normetanephrine and metanephrine are calculated for a unit of increase of 100 µg/die). Abbreviations: ACEi/ARB, angiotensin converting enzyme inhibitors or angiotensin II receptor blockers; BMI, body mass index; CI, confidence interval; CVD, cardiovascular disease; DBP, diastolic blood pressure; DM, diabetes mellitus; FH, family history; OR, odds ratio; SBP, systolic blood pressure.

Covariates	Metabolic syndrome					
	**OR**	**95% CI**	**p-value**	**OR**	**95% CI**	**p-value**
Gender	1.02	0.65-1.59	0.939	1.13	0.72-1.76	0.578
Age	0.99	0.98-1.01	0.445	1.00	0.98-1.01	0.571
Smoking habit	1.26	0.82-1.94	0.293	1.30	0.85-2.00	0.233
FH of CVD	1.32	0.71-2.34	0.363	1.24	0.67-2.20	0.469
No. of antihypertensive drugs	1.33	1.07-1.66	0.010	1.35	1.09-1.68	0.006
eGFR	1.00	0.99-1.01	0.495	1.00	0.99-1.01	0.421
Normetanephrine	1.12	1.01-1.23	0.026	-	-	-
Metanephrine	-	-	-	0.95	0.71-1.22	0.688

Table S2. Logistic regression analysis on the association of metanephrines and covariates with presence of metabolic syndrome, after excluding patients taking drugs acting on the sympathetic system (ORs of normetanephrine and metanephrine are calculated for a unit of increase of 100 μg/die). Abbreviations: CI, confidence interval; CVD, cardiovascular disease; eGFR, estimated glomerular filtration rate; FH, family history; OR, odds ratio.

Covariates	Microalbuminuria					
	**OR**	**95% CI**	**p-value**	**OR**	**95% CI**	**p-value**
Gender	1.71	0.93-3.14	0.084	1.56	0.85-2.89	0.152
Age	1.00	0.98-1.03	0.693	1.00	0.98-1.03	0.710
Smoking habit	1.04	0.56-1.88	0.907	1.00	0.54-1.82	0.998
FH of CVD	1.24	0.45-2.86	0.647	1.30	0.48-3.01	0.573
BMI	0.97	0.91-1.03	0.399	0.97	0.91-1.03	0.423
SBP	0.99	0.97-1.02	0.674	0.99	0.97-1.02	0.599
DBP	1.01	0.98-1.05	0.628	1.01	0.98-1.05	0.635
DM	1.74	0.67-4.00	0.216	1.77	0.68-4.06	0.207
No. of antihypertensive drugs	0.68	0.39-1.13	0.158	0.68	0.38-1.12	0.153
ACEi/ARB	2.00	0.81-5.04	0.135	2.01	0.81-5.12	0.139
Normetanephrine	0.97	0.82-1.11	0.714	-	-	-
Metanephrine	-	-	-	1.21	0.86-1.63	0.224

Table S3. Logistic regression analysis on the association of covariates with presence of microalbuminuria, after excluding patients taking drugs acting on the sympathetic system (ORs of metanephrine are calculated for a unit of increase of 100 μg/die). Abbreviations: ACEi/ARB, angiotensin converting enzyme inhibitors or angiotensin II receptor blockers; BMI, body mass index; CI, confidence interval; CVD, cardiovascular disease; DBP, diastolic blood pressure; DM, diabetes mellitus; FH, family history; OR, odds ratio; SBP, systolic blood pressure.

Covariates	eGFR<60 mL/min/1.73 m^2					
	**OR**	**95% CI**	**p-value**	**OR**	**95% CI**	**p-value**
Gender	0.76	0.43-1.31	0.328	0.86	0.48-1.49	0.588
Age	1.05	1.03-1.07	<0.001	1.05	1.03-1.07	<0.001
Smoking habit	0.85	0.50-1.45	0.563	0.90	0.52-1.53	0.694
FH of CVD	1.41	0.64-2.87	0.369	1.33	0.60-2.71	0.450
BMI	0.97	0.91-1.02	0.255	0.96	0.91-1.02	0.208
SBP	1.02	1.00-1.04	0.027	1.02	1.00-1.04	0.017
DBP	0.99	0.96-1.01	0.290	0.98	0.96-1.01	0.247
DM	1.56	0.74-3.12	0.223	1.50	0.70-3.00	0.274
No. of antihypertensive drugs	1.34	1.02-1.75	0.032	1.36	1.04-1.78	0.026
Normetanephrine	1.02	0.90-1.14	0.696	-	-	-
Metanephrine	-	-	-	0.73	0.47-1.05	0.120

Table S4. Logistic regression analysis on the association of metanephrines and covariates with presence of eGFR<60 mL/min/1.73 m^2, after excluding patients taking drugs acting on the sympathetic system (ORs of normetanephrine and metanephrine are calculated for a unit of increase of 100 μg/die). Abbreviations: BMI, body mass index; CI, confidence interval; CVD, cardiovascular disease; DBP, diastolic blood pressure; DM, diabetes mellitus; eGFR, estimated glomerular filtration rate; FH, family history; OR, odds ratio; SBP, systolic blood pressure.
